# Transcranial Direct Current Stimulation and Migraine—The Beginning of a Long Journey

**DOI:** 10.3390/jcm9041194

**Published:** 2020-04-22

**Authors:** Samar S. Ayache, Moussa A. Chalah

**Affiliations:** 1 EA 4391, Excitabilité Nerveuse et Thérapeutique, Université Paris-Est-Créteil, 94010 Créteil, France; samar.ayache@aphp.fr; 2 Service de Physiologie, Explorations Fonctionnelles, Hôpital Henri-Mondor, AP-HP, 94010 Créteil, France

**Keywords:** migraine, headache, medication overuse headache, tDCS, transcranial direct current stimulation

## 1. Introduction

Migraine, a benign yet disturbing condition, is one of the frequent neurological disorders, affecting up to 15–20% of the worldwide population. This syndrome ranks sixth among the most common causes of disability across the globe [1]. It exerts a drastic impact on the patients’ quality of life and their professional performance [2]. 

From a clinical viewpoint, according to the third edition of the international classification of headache disorders (ICHD-3), migraine is a primary headache disorder and consists of recurrent headache attacks (at least five attacks) of 4–72 h duration [3]. Headaches characteristics should meet at least two of the following criteria—(a) unilateral, (b) throbbing in nature, (c) of moderate to severe intensity, and (d) prone to be worsened by physical activity. Moreover, attacks could be accompanied by other symptoms, like photophobia, phonophobia, nausea, and vomiting. Headaches could be preceded by some warning neurological symptoms, known as aura, and the condition is hence known as migraine with aura. The migraine attack could also start without any preceding phenomenon, and in this case, it is described as migraine without aura, which is found to be more frequent than the former one [3]. 

Two distinct subtypes of migraine have been defined so far, the episodic and chronic migraine, and a cutoff of 15 headache days per month has been established to distinguish between both entities [3]. Moreover, a variant of chronic migraine has been defined, the medication overuse headache (MOH), which occurs in patients with previously existing headache and those who tend to overuse symptomatic headache medications over at least 3 months (ICHD-3 criteria, [3]).

From a pathophysiological perspective, it is now widely accepted that migraine is a complex syndrome and several underlying mechanisms have been described at its origin. For instance, a bulk of literature has described the phenomenon of cortical spreading depression behind the generation of aura in migraineurs. Its occurrence in migraine without aura has been debated for a long time, but recent data support its presence in this subtype. In addition, it is now widely accepted that activation of trigeminovascular pathways, and brainstem and diencephalic nuclei plays a pivotal role in the pathophysiology of migraine [4]. 

Large amount of data has evoked the existence of some excitability changes in the occipital cortex of migraineurs during the interictal period. While some studies have depicted a hyperexcitability state, a decrease in the occipital excitability was documented by few studies. This increase in occipital excitability was recently considered to be an under-inhibition phenomenon and a new term—hyperresponsiveness—was introduced in the last decade. Such a hyperresponsive state is reflected by a lack of habituation to recurrent stimuli, a finding that has been reported on several occasions by neurophysiological and neuroimaging studies [5].

## 2. Therapeutic Approaches 

In addition to the avoidance of potential triggering factors (i.e., sleep deprivation, stress, alcohol consumption, etc.), the management of migraine includes acute abortive medications and, if needed, prophylactic treatments. While the former category aims at alleviating the intensity and/or shortening the duration of the headache episode, the latter tends to decrease the number of headache days per month, improve the patients’ quality of life and decrease the migraine burden. Prophylactic therapy is indicated in case of frequent attacks or resistance to acute treatments. In this perspective, several strategies could be tried, including few pharmacological and non-pharmacological interventions. Medications include beta-blockers, anti-epileptics, antidepressants, calcitonin-gene related peptide monoclonal antibodies, and botulinum toxin injection. Apart from the newly available monoclonal antibodies, for which phase 3 studies and recent meta-analysis have documented promising efficacy [6], the available drugs usually face numerous side-effects and partial satisfaction. These drawbacks have led to the development of numerous non-pharmacological approaches, such as acupuncture [7], chiropractic therapy [8], supraorbital nerve stimulation [9], vagal nerve stimulation [10], greater occipital nerve stimulation [11], psychological interventions [12], and noninvasive brain stimulation [13]. The latter englobes transcranial magnetic stimulation (TMS) and transcranial direct current stimulation (tDCS). These techniques are respectively based on the application of a magnetic field or an electrical current over the scalp, in order to modulate the functioning of neurological circuits. Given the practicality of tDCS, its relative low cost and its possible use as a home-based therapy, recent years have witnessed an increased interest in its use and a growing data on its investigation in several psychiatric and neurological domains, like migraine [13].

## 3. Transcranial Direct Current Stimulation

tDCS consists of delivering a weak current through two sponge electrodes fixed on the scalp and connected to a battery-driven stimulator [13]. It has been established that this intervention exerts its effects through the modulation of resting membrane potential of neural fibers. This modulation depends on the stimulation polarity with anodal and cathodal stimulation leading to a depolarization and a hyperpolarization, respectively [13]. Over the last decade, tDCS has been explored as a preventive therapy in migraine. Few studies have been published so far on this topic [14,15,16,17,18,19,20,21,22,23,24,25,26,27], among which a recent one has investigated the efficacy of this intervention on MOH [25]. While some studies have focused on the stimulation of the occipital (visual) cortex (cathodal in [14,18,19,22,25] and anodal in [17]), which is a key area in migraine pathophysiology, others tested the impact of stimulating the dorsolateral prefrontal cortex (DLPFC) [21,25] (or the frontal pole [24]) (anodal), the primary motor cortex (M1) (anodal in [15,16,20,21,27], cathodal in [26]), and the primary sensory cortex (S1) (cathodal) [26].

The rationale behind choosing the occipital cortex finds its roots in many physiological and functional imaging studies that have reported an altered visual processing and a state of occipital hyperexcitability in migraineurs, during the interictal period. These phenomena have been reflected by a discomfort during the application of repetitive visual stimuli, a decrease in the phosphene threshold and an increase in the occipital metabolism.

The choice of DLPFC was based on a large body of data pointing towards its involvement in pain processing mechanisms [13]. In fact, DLPFC appears to exert a top-down effect on the pain modulating circuits, and stimulation of this area has been found to modulate the activity of other structures, such as the caudate nucleus and the anterior cingulate cortex. Furthermore, imaging and neurophysiological studies have demonstrated that DLPFC is linked to several cortical and subcortical areas, and, therefore, it could be perceived as a carrefour that gives passage to various circuits implicated in emotional-cognitive processes [21]. 

Only one study compared the efficacy of anodal DLPFC stimulation to that of cathodal occipital stimulation [25]. It was performed in patients suffering from MOH and had documented an amelioration of the condition following both approaches, with greater improvement obtained after the occipital intervention. Additional works are required before setting any recommendation on the matter. 

It is worth noting that a recent study aimed to modulate the activity of the frontal cortex through the application of cathodal tDCS [23]; the approach was personalized and the choice of the electrode position depended on the location of what is known as the “cold patch” (the cathode was placed over the frontal cortex ipsilateral to the patch, the reference electrode on the arm). The latter is located on the forehead based on thermographic images; it reflects a hypothermic area suggested to be due to a shunt between the internal and external arteries, and is considered to be specific to migraineurs [23]. Authors have found long-lasting positive effects manifested by a decrease in the number of headache days, number and duration of migraine attacks, analgesics consumption, and pain intensity. It should be kept in mind that this individualized mononcephalic approach, although interesting, has not been tested in other works, and further proofs on its utility are required. 

As mentioned above, apart from DLPFC and occipital cortices, M1 has attracted the attention of some researchers that perceived migraine as a chronic pain condition and decided to act on the pain matrix through the application of anodal stimulation over this cortical area; which is widely adopted in other chronic pain conditions [13]. Anodal M1 stimulation has led to some beneficial effects on migraine, as manifested by a decrease in the number of attacks [16], reduction of pain intensity [15,21], improvement of the quality of life [21], and decline of painkillers consumption [21]. A comparison between anodal DLPFC and anodal M1 tDCS was performed by Andrade and colleagues, who depicted positive effects following both interventions, with superior effects observed after the former one. These findings merit testing in further large-scale studies. 

Interestingly, instead of anodal tDCS, one study applied cathodal tDCS over M1 or S1, both of which take part in the pain matrix [26]. Both conditions yielded clinical benefits, which might be due to the restoration of the previously described habituation phenomenon or the modulation of pain and sensory thresholds [26]. This observation is worth exploring in future works. 

## 4. Conclusions and Perspectives

The aforementioned works support the utility of tDCS in the management of migraine and offer a glimmer of hope for patients suffering from this debilitating disease. However, despite these promising results, studies should be interpreted with caution since the majority of them are pilot studies, being performed in a small sample size, with some having adapted an open-label design. Furthermore, their setup showed a great variation in terms of stimulation duration (10 vs. 15 vs. 20 min), electrode polarity (cathodal vs. anodal), current intensity (1 mA vs. 2 mA), cortical target (occipital vs. DLPFC vs. M1 vs. S1), stimulation side (left vs. right), and reference electrode (various cephalic vs. extracephalic positions). The number of stimulation sessions also differed across studies (3–22 sessions) and needs to be considered when designing future works. 

In addition, strategies used to locate the cortical sites differed among the available reports. For instance, the location of M1 corresponded to the hotspot determined by using TMS or was defined according to the 10–20 international electroencephalographic (EEG) system. Moreover, patients’ profile varied across studies, with some of them allowing pain medication intake, while the remaining works considering previous medications intake an exclusion criterion. Furthermore, in some studies only patients having migraine without aura were included, in others the type was not specified, or both types were accepted (migraine with aura and migraine without aura). The outcomes also varied across the studies (e.g., number or duration of attacks, pain intensity, pills consumption, headache impact on quality of life).

For all these reasons, several questions remain unanswered and additional research is obviously needed in order to define the most appropriate cortical site to stimulate, the most efficient set-up to adapt, and the most adequate stimulation duration and rhythm to offer to this clinical population. tDCS studies including neurophysiological (i.e., evoked potentials, EEG, autonomic nervous system measures) and neuroimaging modalities (i.e., functional brain MRI) might allow us to understand the neural underpinning of clinical responses in the context of migraine (i.e., changes in regional activation pattern, resting-state functional connectivity, cortical excitability, EEG rhythms, or autonomic nervous system activity). In addition, performing neuropsychological evaluations would allow us to identify potential psychological and cognitive factors that have been associated with migraine in some studies (i.e., presence of anxiety/depression, specific coping strategies or personality traits, and cognitive manifestations) [28,29,30], and might serve as predictors for tDCS response. Once all these issues are addressed, researchers can open the way to home-based tDCS, notably, the safety of this approach has been proven in other settings and its place in the treatment of various neuropsychiatric diseases is currently under exploration by numerous teams around the globe. In addition, this technique could be coupled with non-pharmacological interventions, like other non-invasive stimulation options (e.g., trans-spinal direct current stimulation and TMS [27,31,32]), or psychotherapies (e.g., cognitive-behavioral therapy, interventions including mindfulness and educational components) [33], with the latter combination having a particular utility in the management of anxiety and depression frequently encountered in migraineurs.
